# Corrosion Resistance of Al–CNT Metal Matrix Composites

**DOI:** 10.3390/ma14133530

**Published:** 2021-06-24

**Authors:** Vladimir V. Popov, Alla Pismenny, Natalya Larianovsky, Anna Lapteva, Daniel Safranchik

**Affiliations:** 1 Institute of Metals, Technion R&D Foundation, Haifa 3200003, Israel; allap@trdf.technion.ac.il (A.P.); natalyal@trdf.technion.ac.il (N.L.); DaniS@trdf.technion.ac.il (D.S.); 2 Department of Regional Economics, Innovation Enterprise and Security, Ural Federal University, 620002 Ekaterinburg, Russia; annalapteva@mail.ru

**Keywords:** aluminium metal matrix composites, carbon nanotubes, CNT, corrosion

## Abstract

The design of aluminium–graphite metal matrix composites (MMCs) with advanced mechanical properties and high corrosion resistance is in demand for aerospace, transportation, and industrial applications. Breakthroughs in this field are limited due to the tendency of aluminium–graphite MMCs to corrode. In the present research, aluminium-based MMCs were produced by a relatively novel combined two-staged method. Multiwall carbon nanotubes (MWCNTs) were added into molten Al1070 and processed by high-pressure die casting followed by cyclic extrusion. For the composites produced by this method, it was previously demonstrated that mechanical properties are improved in comparison with pure aluminium alloys. In the current study, the manufactured Al–MWCNT composites were investigated by electrochemical tests (such as open circuit potential), potentiodynamic tests, linear polarization tests, and electrochemical impedance spectra to understand the corrosion resistance of the obtained composite material. The experimental testing of the corrosion resistance of Al–MWCNT MMCs showed that due to the advantages of the fabrication method, the addition of CNTs to aluminium does not cause a radical decrease of corrosion resistance.

## 1. Introduction

Metal matrix composites (MMCs) are of great interest to leading industries for their various applications in, for example, aerospace, transportation, defence, construction, and industry. These materials are developed for their unique combination of physical and mechanical properties, such as being lightweight, having mechanical strength, and having chemical and corrosion resistance. The control and prediction of corrosion issues are crucial for many industries [1,2], especially for aerospace applications [3,4,5].

Graphite–aluminium MMCs are already well-known materials for advanced industrial applications [6]. However, these MMCs are known as typically prone to corrosion. The corrosion resistance of the metal matrix composites is strongly affected by galvanic corrosion at the interfaces between the metallic matrix and its reinforcements [7]. By now, no significant progress has been achieved in the design of such MMCs with enhanced mechanical properties and sufficient corrosion resistance [7]. 

The problematic issue in the fabrication of aluminium-based MMCs is that alloying additions may reduce the corrosion resistance of the aluminium matrix [8]. It should be taken into account that the corrosion behaviour of graphite–aluminium composites may be strongly related to the selected production method used for their fabrication and the type of carbon-based additives [3,9].

The most typical fabrication methods for Al-based MMCs include liquid phase techniques (stir casting, strip casting, arc/laser melting, etc.), powder metallurgy (ball-milling, compaction, mixing, etc.), and deformation techniques (rolling, extrusion, etc.) [10].

The MMCs with an aluminium matrix reinforced with tungsten carbide (WC) and titanium carbide (TiC) particles are described in [11]. In this work, the authors used submicron-sized WC and TiC particles to reinforce Al1050 in a two-step method: mixing additives with fluxing salt followed by introducing this mix into the molten aluminium alloy. Melting was accompanied by intensive stirring to avoid agglomeration of carbides and to promote homogeneous dispersion. Molten aluminium with induced carbides was poured into moulds [11]. The authors declared that the corrosion behaviour of the obtained MMCs was determined by the corrosion resistance of the selected aluminium alloy. Moreover, carbides and aluminides in the aluminium matrix often prevented intergranular corrosion [11]. Another paper reported Al-based MMCs with SiC reinforcement designed by strip casting [12]. The MMC system that had a greater hardness had better corrosion resistance, but still lower than that of the initial aluminium alloy [12]. Shimizu et al. investigated the corrosion resistance of Al-based metal matrix composites prepared with various additives: carbon fibres, alumina fibres, and silicon carbide whiskers [9]. The authors showed that the corrosion potential for MMCs produced with carbon fibres, alumina fibres, and SiC whiskers was rather the same as for pure Al 6061. However, it was concluded that the presence of carbon additives might be more subjected to pitting [9]. Zakaria showed that Al/SiC MMCs could be fabricated by powder metallurgy methods [13]. The author tested the impact of the fraction size of SiC additives on the corrosion behaviour of the MMCs. It was found that an increase in the amount of SiC in MMC, and the reduction of its fraction size, resulted in corrosion resistance improvement [13].

The significant contribution of the publications cited above and displayed in Table 1 is devoted to the fabrication of MMCs with additives having a fraction size from several to tens of microns. The introduction and dispersion of nano-sized additives, such as carbon nanotubes (CNTs), is a challenging issue, especially by liquid phase metallurgy [14]. However, there were attempts to fabricate aluminium-based composites with CNTs by the rolling technique [15]. The obtained results showed the scalability of the fabrication method and improvement of mechanical properties, but the corrosion behaviour was not evaluated [15]. The main issue in producing Al-based composites containing CNTs by liquid phase metallurgy is to mix them homogeneously in molten aluminium. It is problematic since the CNTs tend to rise to the surface without being mixed because of their poor wetting properties and great difference in their density compared with that of Al [16].

**Figure 1:**
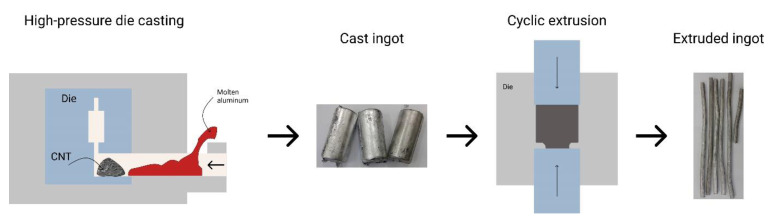
Workflow of HPDC-CE fabrication method.

A combined two-staged method was developed to overcome these issues [14]. The authors developed a method of fabrication of metal matrix composites in which multiwall carbon nanotubes (MWCNTs) were inline dispersed in the Al matrix [14]. Figure 1 shows the method, which includes two main steps. The first step is high-pressure die casting (HPDC) of aluminium, where the MWCNTs are added to the short sleeve of the HPDC furnace prior to aluminium incorporation. In the second step, the obtained cast ingot passes ten cycles of extrusion (CE) to break up agglomerated MWCNTs clusters and to align them in the direction of extrusion.

The introduction of carbon nanotubes in aluminium and their distribution have a crucial effect on the electrical and mechanical properties of the resulting composites [22,23]. Accordingly, it is logical to expect an effect on their corrosion resistance. Therefore, it was important in the present study to determine how the developed method of manufacturing aluminium composites using high-pressure die casting and cyclic extrusion improves or degrades the initial corrosion resistance of the aluminium alloy.

The novel HPDC-CE method used in this research enables the manufacturing of MMCs with a significant increase in mechanical strength [14]. In the present research, it was crucial for us to demonstrate that the proposed fabrication method allows reinforcement of the material without significant degradation of corrosion resistance. We investigated the corrosion behaviour of aluminium-based MMCs produced by this HPDC-CE method and compared them with a pure aluminium 1070 alloy.

The current research demonstrates how the novel production HPDC-CE method affects the corrosion resistance properties of Al–MWCNT composites. 

## 2. Materials and Methods

**Figure 2:**
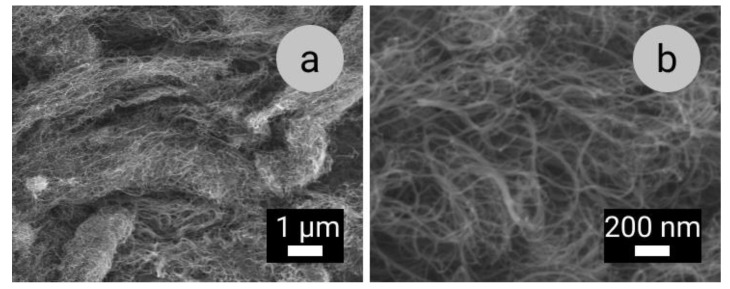
Scanning electron microscopy images of as-received MWCNTs at: (**a**) lower (10.00 K) and (**b**) higher (50.00 K) magnifications.

The raw materials used were aluminium alloy Al 1070 and MWCNTs (Nanocyl SA, Sambreville, Belgium) of 90% purity (see Figure 2 and Table 2). The experimental Al–MWCNT MMCs were prepared at the Israeli Institute of Metals by a high-pressure die casting technique followed by cyclic extrusion as described in [14]. 

Three Al-based groups of samples were characterized:

- A—pure Al 1070;
- B—Al 1070 with 0.25 wt.% MWCNTs;
- D—Al 1070 with 0.5 wt.% MWCNTs.

All groups of samples were produced by high-pressure die casting followed by 10 cycles of extrusion according to [14]. The examined surface of the samples was perpendicular to the extrusion direction. For each group of samples, between 3 and 5 surfaces were examined.

High-resolution SEM images were obtained with a TESCAN-MIRA3 high-resolution field emission gun (FEG) scanning electron microscope (Zeiss Ultra, Oberkochen, Germany), equipped with the AZtecHKL standard package.

The HPDC-CE samples were cut and polished for microstructural examination using standard SiC grit papers and required emulsions. The final grid paper was of 4000 mesh for 60 s. The samples had a diameter of 10 mm and height 8 mm. The samples for corrosion testing were cut and polished strictly under the same conditions.

**Figure 3:**
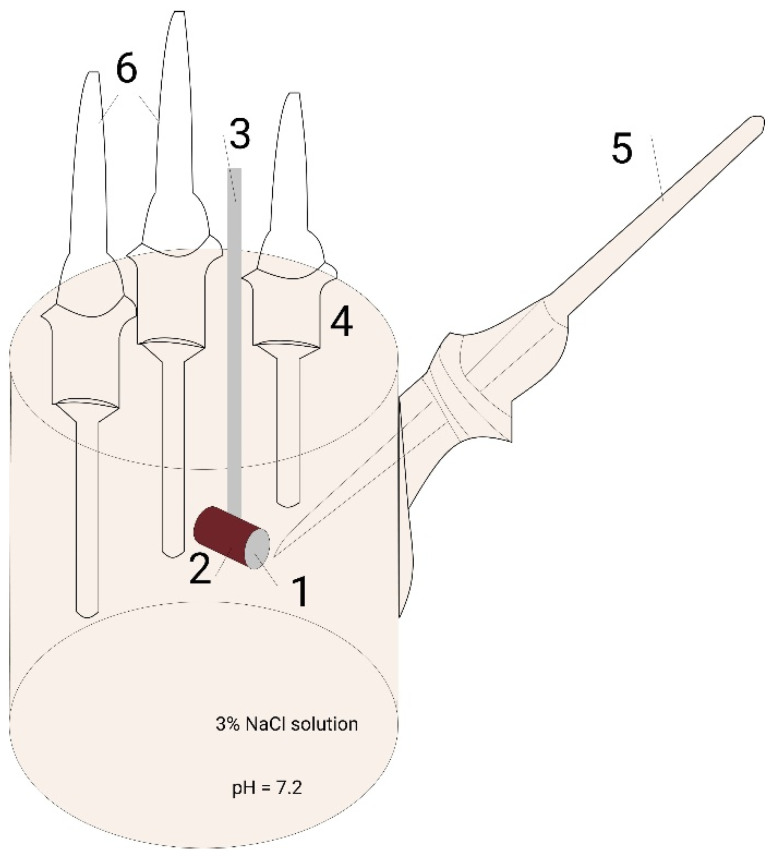
Experimental scheme of corrosion potential testing by potentiodynamic anodic polarization technique, where 1: tested sample surface (working electrode); 2: isolate sample with only one tested surface; 3: sample holder; 4: testing glass with 3% NaCl solution with pH = 7.2; 5: Calomel electrode with probe membrane; and 6: auxiliary electrode holders.

Corrosion measurements were conducted for the pure aluminium specimen as a reference and for the HPDC-CE produced MMCs using the electrochemical cyclic polarization technique according to [24]. The scheme of the corrosion resistance potentiodynamic measurement is shown in Figure 3. Electrochemical measurements were performed at room temperature in a 3% NaCl solution with pH = 7.2 before testing. The saturated Calomel electrode (sat’d KCl) was used according to the standard [24]. The parameters of electrochemical measurements, including repeatable electrochemical impedance spectroscopy, are shown in Table 3.

## 3. Results

Figure 1 shows that the used MWCNTs were strongly agglomerated, and that was why several cycles of extrusion were required to break the agglomerates and to disperse them homogeneously in the aluminium matrix.

**Figure 4:**
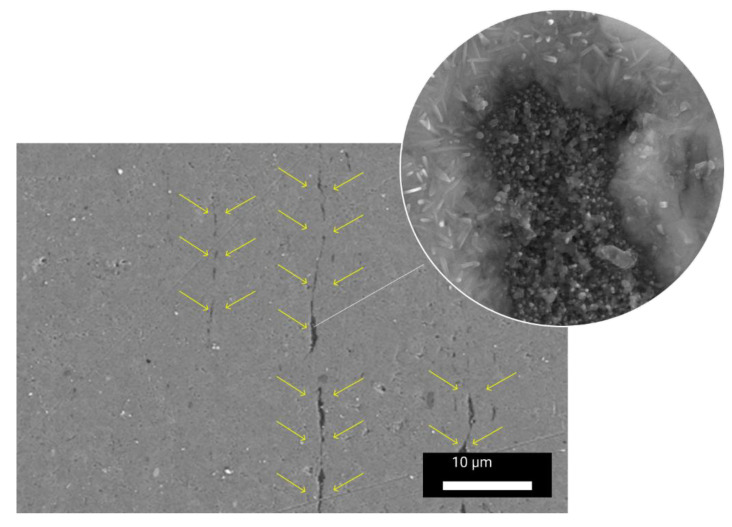
HRSEM images of the Al − 0.5 wt.% CNT composites after 10 cycles of cyclic extrusion. MWCNT-rich clusters are signified by arrows.

It has been found that the amount of MWCNTs that can be introduced into an aluminium matrix by the two-staged HPDC-CE method is limited. The introduction of limited amounts of MWCNTs using the HPDC-CE method results in improving the mechanical strength. The ultimate strength was observed to be 94 MPa for 1070 aluminium, 126.8 MPa for Al + 0.25% MWCNTs, and 132.2 MPa for Al + 0.5%MWCNTs after the same ten number of extrusion cycles. The yield strength was also increased from 64 MPa for the initial 1070 alloy, to 99.2 MPa for Al + 0.25% MWCNTs, and to 104 MPa for Al + 0.5% MWCNTs [14]. The improvement of the mechanical performance of the composites with the addition of MWCNTs is due to the superior mechanical properties of these carbon nanotubes. The increase of the MWCNTs amount to higher than 0.5% leads to a reduction in mechanical properties [14,25,26]. Such behaviour may be attributed to the presence of MWCNT clusters that can be easily demonstrated by SEM (Figure 4).

It can also be seen in Figure 4 that the CNT clusters have a specific linear character. Due to the cyclic extrusion process, it was possible to reduce the CNT clusters up to 200 nm. They are aligned in the direction of extrusion and look like parallel passes in cylindrical samples. This makes possible the properties of anisotropy in line with the extrusion direction and in the surfaces perpendicular to it.

**Figure 5:**
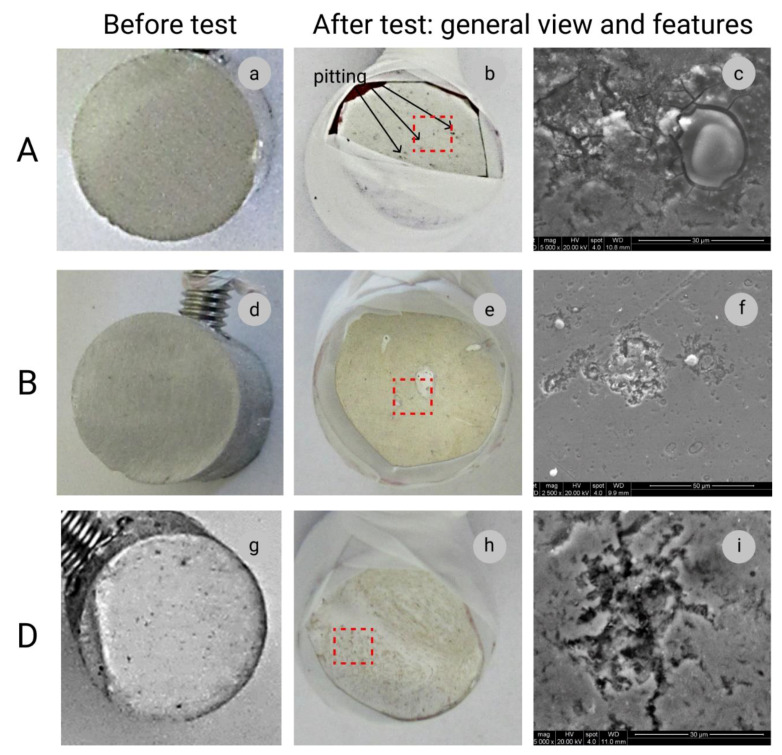
Sample A—pure aluminium 1070 produced by die casting and direct extrusion: (**a**)—the test surface before the test; (**b**)—image of the sample’s surface after the test; (**c**)—SEM image of specific features on the sample’s surface because of corrosion. Sample B—Al-based composite sample with 0.25 wt.% of MWCNTs: (**d**)—the test surface before the test; (**e**)—image of the sample’s surface after the test; (**f**)—SEM image of specific features on the sample’s surface because of corrosion. Sample D—Al-based composite sample with 0.5 wt.% of MWCNTs: (**g**)—the test surface before the test; (**h**)—image of the sample’s surface after the test; (**i**)—SEM image of specific features on the sample’s surface because of corrosion.

Sample A—pure aluminium has been tested to demonstrate the corrosion resistance of aluminium as a reference. Figure 5a shows the initial surface for the test, and Figure 5b shows the surface after testing. The light pitting corrosion of the sample A surface can be observed. Figure 5c shows areas of cracking of the material surface in the pitting zone. Figure 5d,g shows the initial surfaces of the samples B and D, correspondingly, before tests. For both composite samples, the same pitting behaviour can be observed (see Figure 5e,h). At a higher magnification (Figure 5f,i), it can be seen that in composite samples, pitting looks more aggressive in CNT clusters areas. This can be explained by the fact that Al-based composites are more prone to pitting than pure aluminium alloys without reinforcement [7,27]. It is also a well-known fact that pitting is one of the main corrosion types for aluminium alloys and the composites based on them [17,28].

**Figure 6:**
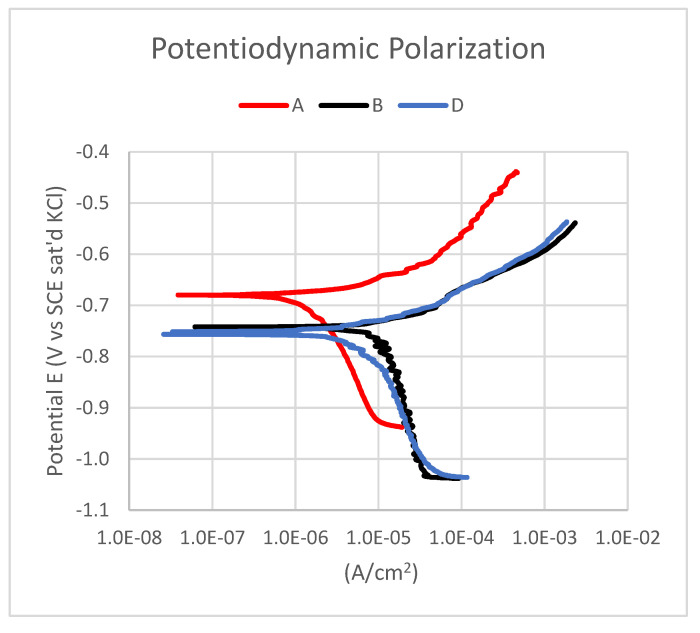
Potentiodynamic electrochemical measurement of sample A—pure aluminium 1070; sample B—Al 1070 + 0.25 wt.% MWCNTs; and sample D—Al 1070 + 0.5 wt.% MWCNTs.

The potentiodynamic polarization curves of the aluminium 1070 alloy and the Al–MWCNT composites are shown in Figure 6. The corresponding Tafel results are shown in Table 4. The potentiodynamic polarization curves of all samples were basically the same in shape, which indicates that these two materials have similar electrochemical corrosion behaviour in 3% NaCl solution. The polarization curves do not show passive regions; therefore, the corrosion type of the two materials in the 3% NaCl solution is an active corrosion process [17]. However, according to the fitting results of the polarization curves, the composite samples have a lower corrosion potential and almost the same corrosion current density compared with those of the 1070 aluminium alloy. Due to the pitting type of corrosion in all groups of samples, the corrosion rate could not be calculated.

**Figure 7:**
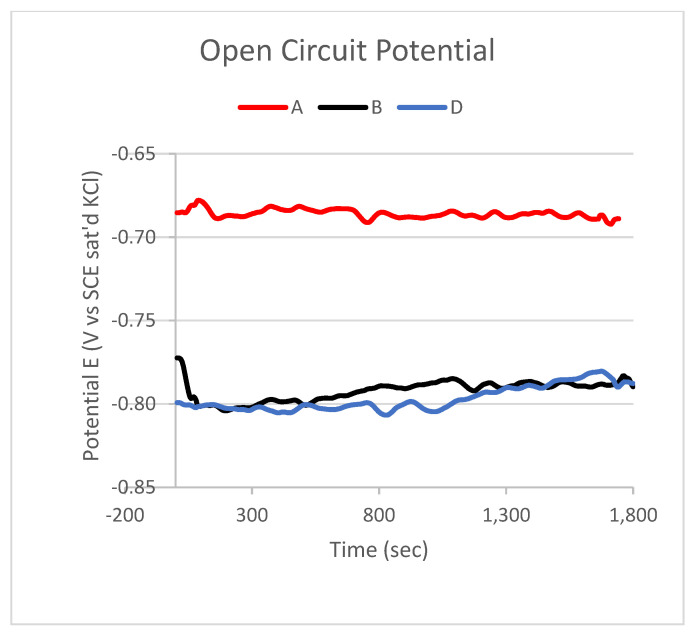
Open circuit potential measurement of sample A—pure aluminium 1070; sample B—Al 1070 + 0.25 wt.% MWCNTs; and sample D—Al 1070 + 0.5 wt.% MWCNTs.

Figure 7 shows the open circuit potential (OPC) linear polarization curves. The OPC confirms the results of the potentiodynamic data: the corrosion potential of composite samples is lower than it is for the 1070 alloy sample. It can also be concluded that sample D, with a concentration of MWCNTs two times higher than sample B, has the same corrosion potential.

**Figure 8:**
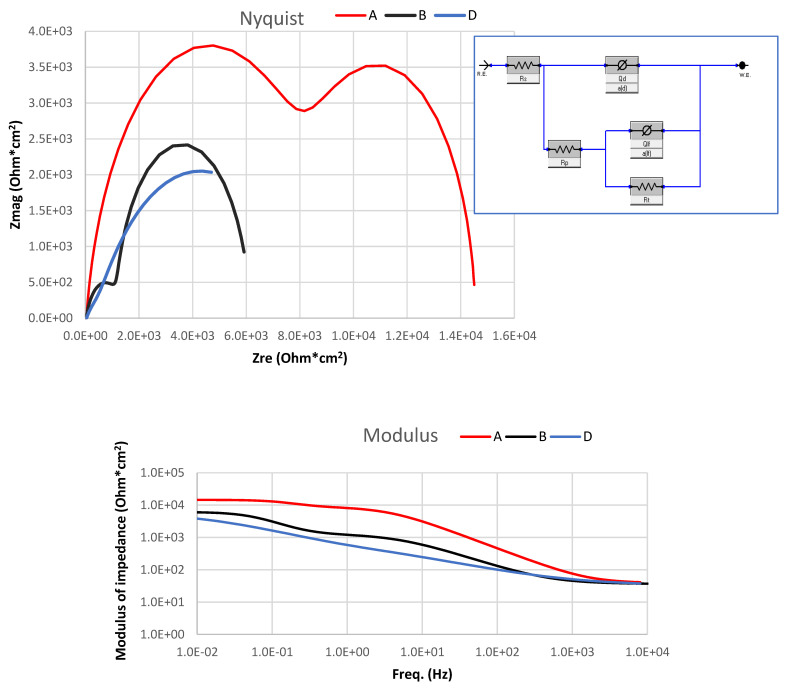

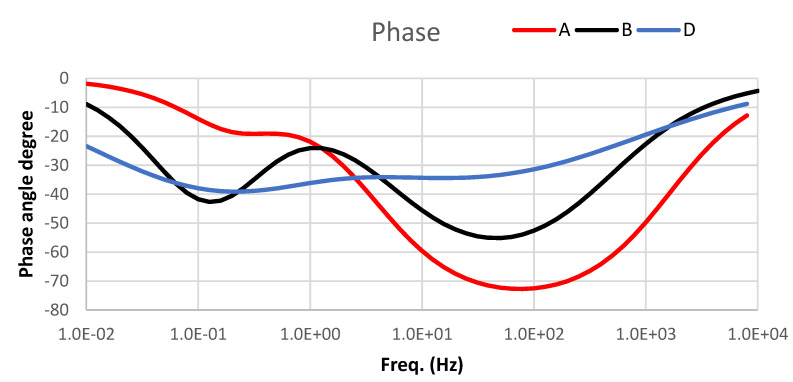
Electrochemical impedance spectra of sample A—pure aluminium 1070; sample B—Al 1070 +0.25w.% MWCNTs; and sample D—Al 1070+0. 5w.% MWCNTs.

Figure 8 shows the electrochemical impedance spectrum (EIS) aiming to provide more information about the mechanism of the corrosion reaction of the 1070 alloy (sample A) and Al–MWCNT composites (samples B and D). In the Nyquist EIS, one can observe two capacitive loops at high and intermediate frequencies and an inductive loop at low frequencies. The high-frequency loop is associated with the electron transfer process in the interface metal/oxide [29]. At low frequencies, because of the high dispersion, there is no steady state in the impedance diagram, and it is challenging to observe the inductive loop. Some authors suggest significantly increased stabilization times up to 96 h to reach steady state [30]. All three spectra demonstrate the inductive character in the Nyquist plot. The inductive behaviour is attributed to the active nucleation of pits [31]. The impedance spectra of the 1070 alloy and the composite samples have capacitive loops at high frequencies. However, the capacitive loop of sample A (pure 1070 alloy) shows a larger diameter than the loops of the composites (samples B and D). The high-frequency loop is related to the oxide film on the aluminium surface under the corrosion process, and the large diameter of the capacitive reactance indicates that the impedance of the oxide film is higher. The inductive loops of all samples are affected by Cl^−^ adsorption caused by the destruction of the oxide film on the surface of the sample [17,32]. The equivalent electrical circuit fitting results are shown in Table 5. Table 5 shows that an increase of MWCNT content results in a reduction of polarization resistance (R_p_).

The quantitative data on corrosion potential measurements for all the three groups of samples are summarized in Table 4.

The results collected in Table 4 and the graphs in Figure 6, Figure 7 and Figure 8 demonstrate that the MWCNT content does not radically affect the corrosion resistance of the produced Al-based MMCs. However, the corrosion potential value for pure aluminium is −680 mV, and for the obtained composite samples, it is −745 mV relative to the SCE saturated calomel (saturated KCl), that is, the potential for composites is lower by 65 mV with the MWCNT content. The developed Al-based MWCNT MMCs have the same corrosion mechanism and comparable corrosion resistance as the initial aluminium alloy. 

## 4. Conclusions

In the present study, the corrosion behaviour of Al-based metal matrix composites (MMCs) containing multiwall carbon nanotubes (MWCNTs) has been investigated. The Al-MWCNT composites were fabricated by the two-stage melt-based HPDC-CE method.

The development of new hybrid/mixing/combined techniques is still underway and requires the improvement of CNT dispersion in the aluminium matrix. This might result in high mechanical performance and sufficient corrosion resistance.

In the researched specimens, the MWCNT clusters were observed in the extrusion direction. However, cyclic extrusion facilitates the MWCNT alignment in this direction, which is accompanied with the strengthening of the MMCs. In spite of the clusters/agglomerates issue, the combination of high-pressure die casting and cyclic extrusion for manufacturing Al–MWCNT metal matrix composites promises many benefits, namely the effectiveness of the introduction of MWCNTs, inline alignment of the MWCNTs, low waste of material, mechanical strength improvement, and reasonable corrosion resistance properties.

The inclusion and distribution of carbon nanotubes in aluminium using the proposed fabrication technique changes the corrosion potential of the composites in comparison with 1070 alloy. The tests show the same pitting mechanism of corrosion for all examined samples. The produced Al–MWCNT composites that have a higher mechanical strength are characterized by a more negative corrosion potential. Still, this can be considered as a positive result for the further development of the fabrication method of Al-based MWCNT composites. The presented experimental findings illustrate a pathway to new applications where lightweight materials with mechanical strength and reasonable corrosion resistance are of great demand, such in the aerospace industry, the gas and oil industry, and defence-oriented products.

## Figures and Tables

**Table 1:** Al-based MMCs fabricated by various techniques with various reinforcement additives.

Aluminium Alloy	Additive	Fabrication Method	Aimed Application	Ref.
AA1050	WC, TiC	Mixing and casting	Aerospace, defence, automotive	[11]
AA6063	SiC, ash particles	Stir casting	Aerospace	[12]
AA6063	TiC, Al_2_O_3_	In situ reaction and “near-liquid casting method”	Aerospace, automobile	[17]
AA6061	Fly ash	Stir casting	Aerospace	[18]
Pure Al	Al_2_O_3_, SiC, graphite particles	Stir casting	High-tech construction applications	[19]
AA7178	ZrB_2_	Stir casting	Aerospace, automobile	[20]
AA6061	C fibres, Al_2_O_3_ fibres, SiC whiskers	Squeeze casting	Aerospace	[9]
Pure Al	Al_2_O_3_	High-energy ball milling	Aerospace, automobile	[21]
Al powder of 99.8% purity	SiC	Mixing and compaction	Aerospace, transportation	[13]
AA1100-H12	MWCNTs	Ultrasonic assembly & rolling	Aerospace, industrial applications	[15]
AA1070	MWCNTs	HPDC-CE	Aerospace, industrial applications	[14]

**Table 2:** Specification of MWCNTs by Nanocyl SA, Sambreville, Belgium.

Type	Outer Diameter, nm	Length, μm	Purity, wt.%	Surface Area, m^2^/g
NC7000 Nanocyl SA	9.5	1.5	90	250–300

**Table 3:** Testing conditions and parameters.

Potentiodynamic Test							
Test cell	Solution	Temperature	Er equilibrium	Potentiostat	Auxiliary electrodes	Reference electrode	
Glass bulb with 0.8 L of test solution	0.1 N NaCl	25 ± 1 °C	30 min	Gamry Reference 3000	Graphite (X1)	Saturated Calomel Electrode (sat’d KCl)—SCE	
**Linear polarization parameters**							
Initial E	Final E	Sample area			Polarization scan rate		
−250 mV	+250 mV	A-0.63 cm^2^	B-0.63 cm^2^	D-0.47 cm^2^	1 mV/s		
**Impedance potentiostatic parameters**							
Initial freq. (kHr)	Final freq. (mHr)	Sample area			DC voltage (V) = Eoc		
100	10	A-0.63 cm^2^	B-0.63 cm^2^	D-0.47 cm^2^	Eoc(A) = −680	Eoc(B) = −744	Eoc(D) = −746

**Table 4:** Potentiodynamic measurement results for selected group of samples.

Sample	Material	Approximate Surface Area (cm^2^)	E_cor_ (mV) after 0.5 h vs. SCE	E_cor_ (mV) after 1 h vs. SCE	I_cor_ (µA)	pH	
						Before Test	After Test
A	Al1070	0.63	−689	−680	0.03	7.05	6.54
B	Al1070 + 0.25% (*w*/*w*) MWCNTs	0.63	−789	−44	0.06	7.0	6.74
D	Al1070 + 0.5% (*w*/*w*) MWCNTs	0.47	−787	−746	0.04	7.1	6.7

**Table 5:** Impedance spectrum fitting results of the initial 1070 alloy and the composite samples.

Sample	Surface	Rs	Qd	a(d)	Rp	Qlf	a(lf)	Rt
	(cm^2^)	(Ohm*cm^2^)	(S*sec^a/cm^2^)		(Ohm*cm^2^)	(S*sec^a/cm^2^)		Ohm*cm^2^
A	0.63	39	7.12 × 10^−6^	0.89	9.00 × 10^3^	1.79 × 10^−4^	1	5.51 × 10^3^
B	0.63	36	4.99 × 10^−5^	0.80	1.34 × 10^3^	5.45 × 10^−4^	1	4.78 × 10^3^
D	0.47	32	3.60 × 10^−4^	0.54	5.64 × 10^2^	5.26 × 10^−4^	0.5	2.54 × 10^4^

## Data Availability

The data presented in this study are available on request from the corresponding author. The data are not publicly available due to confidentiality.
